# The choice of adjuvant radiotherapy in pancreatic cancer patients after up-front radical surgery

**DOI:** 10.1371/journal.pone.0317995

**Published:** 2025-01-24

**Authors:** Xia Xiao, Pei Huang, Xiao-Ting Xu

**Affiliations:** 1 Department of Oncology, Wuxi No.2 People’ s Hospital, Jiangnan University Medical Center, Wuxi, Jiangsu Province, China; 2 Department of Radiation Oncology, The First Affiliated Hospital of Soochow University, Suzhou, Jiangsu Province, China; Universitá Sapienza di Roma, ITALY

## Abstract

**Background:**

The role of adjuvant radiotherapy in pancreatic cancer following radical surgery remains a subject of of controversy. This study aimed to more accurately screen pancreatic patients who benefit from adjuvant radiotherapy.

**Methods:**

Clinicopathologic characteristics of patients with resectable pancreatic cancer were collected from the Surveillance, Epidemiology, and End Results (SEER) database (2004–2015). Univariate and multivariate analyses were applied to identify prognostic factors affecting patient survival. All the patients were divided into two groups, one receiving radiation and the other not. Selection bias were reduced by propensity-score matching (PSM). Kaplan-Meier analysis was used to estimate overall survival (OS) and cancer-specific survival (CSS) between the two groups.

**Results:**

Within 7097 patients, 2276 received adjuvant radiotherapy (external beam radiation), and 4821 did not. Multivariate analysis revealed that race, age, median income, sex, year of diagnosis, American Joint Committee on Cancer (AJCC) T stage, N stage, scope region lymph surgery, chemotherapy, and radiotherapy were independent predictors for overall survival of all the patients (all p < 0.05). After PSM, a total of 4304 patients were included. There was no OS and CSS benefit of radiotherapy compared with no-radiotherapy (all p > 0.05). Among patients with N_1_ stage, the radiotherapy group exhibited a median overall survival (mOS) of 21 months (95% CI, 19.82 to 22.18), while the non-radiotherapy group showed a slightly lower mOS of 18 months (95% CI, 16.88 to 19.12). Similarly, in terms of median cancer-specific survival (mCSS), the radiotherapy group demonstrated a mCSS of 22 months (95% CI, 20.79 to 23.21), whereas the non-radiotherapy group had a slightly shorter mCSS of 19 months (95% CI, 17.81 to 20.19). Radiotherapy reduced the all-cause mortality rate and cancer-specific mortality rate among patients with the N_1_ stage and T_4_ stage (all p < 0.05). In contrast, the patients in the radiotherapy group with the N_0_ stage (mOS, 28 months versus 34 months; mCSS, 30 months versus 41months), or primary focus on the body and tail of the pancreas (mOS, 23 months versus 29 months; mCSS, 25 months versus 32 months), or T_1_ stage (mOS, 36 months versus 113 months; mCSS, 36 months versus 104 months) exhibited a higher all-cause mortality rate and cancer-specific mortality rate compared to those without radiotherapy (all p < 0.05). Subgroup analysis indicated N_1_ stage pancreatic cancer patients with T_2-4_ stage, primary focus on the head of the pancreas, young age of onset, and combination chemotherapy were in favor of the adjuvant radiotherapy group (all p < 0.05).

**Conclusions:**

Our analysis demonstrates that adjuvant radiotherapy may be beneficial for N_1_ stage (N+) pancreatic cancer patients who have undergone up-front radical surgery with T_2-4_ stage, primary focus on the head of the pancreas, young age of onset, and receiving combination chemotherapy. However, radiotherapy needs to be used with caution in patients with T_1_ stage, N_0_ stage (N-), or primary focus on the body and tail of the pancreas. These findings may contribute to the development of personalized selection criteria for adjuvant radiotherapy in post-surgical pancreatic cancer patients.

## 1. Introduction

Pancreatic cancer is a highly fatal disease with an approximately 10% 5-year survival rate all over the world. Its incidence is rising every year, making it an increasingly common cause of cancer-related deaths. Patients typically present with advanced disease due to lack of or vague symptoms when the cancer is still localized [1,2]. Surgery has remained the only curative treatment of pancreatic cancer for over a decade [1]. Unfortunately, in resectable pancreatic cancer patients who received surgery treatment, the prognosis was not very satisfactory, and local recurrence events ranged from 20% to 50% [3]. Thus, the treatment concept of pancreatic cancer has changed from simple surgery to comprehensive treatment.

The role of radiotherapy in improving the survival of patients with pancreatic cancer after radical surgery remains ambiguous. The Gastrointestinal Tumor Study Group (GITSG) demonstrated a significant survival advantage for patients who received adjuvant combined radiation and chemotherapy following curative resection of pancreatic cancer. The median survival was 10.9 months in the control group compared to 21.0 months for those who randomly received the treatment. This survival benefit in the treatment group might be due to chemotherapy or radiotherapy or chemoradiotherapy [4]. The EORTC trial, a larger-powered study designed to validate the smaller GITSG trial results, used a similar adjuvant therapy, except it did not include chemotherapy. Patients with T_1-2_N_0-1a_M_0_ pancreatic head or T_1-3_N_0-1a_M_0_ periampullary cancer were included in the EORTC trial while pancreatic patients following curative resection were studied in the GITSG study. Unlike the GITSG trial, the EORTC trial did not find a statistically significant benefit to adjuvant chemoradiation [5]. However, through further analysis of pancreatic cancer subgroups, with one-sided log-rank test would have been used, the EORTC trial would have suggested a benefit to adjuvant chemoradiotherapy. Together with the results of the GITSG trial, there is vital phase III evidence that patients may benefit from adjuvant chemoradiotherapy [6]. In contrast, European ESPAC-1 trial concluded that adjuvant chemotherapy has a significant survival benefit in patients with resected pancreatic cancer, whereas adjuvant chemoradiotherapy has a deleterious effect on survival. This study has been questioned for grouping irregularity and not according with random selection [7]. The contradiction of the conclusions from different studies may be due to the lack of sufficient enrollment patients, no further screening of enrolled population, inconsistencies in treatment regimens and the differences in statisical methods. Recent retrospective analysis suggests that adjuvant radiotherapy may not be associated with an OS benefit for all patients, but for the subgroup with high risk factors, such as T_4_, N+ or R+ resection [8–10]. Based on NCCN guidelines, adjuvant radiotherapy was recommended in pancreatic cancer patients with N+ and R+ resection [11].

The conflicting conclusions from various studies highlight the need for more precise screening to identify patients who may benefit from adjuvant radiotherapy. Currently the role of adjuvant radiotherapy in pancreatic cancer after up-front radical surgery remains unclear. This retrospective study, based on a large-scale population database, aims to evaluate the efficacy of adjuvant radiotherapy for resectable pancreatic cancer after up-front radical surgery.

## 2. Materials and methods

### 2.1 Patient selection

The account was registered with the official SEER site and the SEER*Stat software was downloaded. The user ID is “19331-Nov2021”. The patient data for this study were extracted from the SEER database, which includes comprehensive cancer statistics for the U.S. population. The database contains detailed incidence and demographic info, such as race, age, marital status, median income, sex, year of diagnosis, primary site, T stage at diagnosis, N stage at diagnosis, the procedure of removal, biopsy, or aspiration of regional lymph nodes performed during the initial work-up or first course of therapy at all facilities (scope region lymph surgery), chemotherapy, radiotherapy, number of tumors and survival information. Patients were diagnosed as pancreatic cancer according to the Sixth Edition of the American Joint Committee on cancer (AJCC) staging manual (T_1_: localized within the pancreas, with diameter ≤ 2cm, T_2_: localized within the pancreas, with diameter > 2cm; T_3_: extends beyond the pancreas but not involving the celiac axis or superior mesenteric artery; T_4_: involving the celiac axis or superior mesenteric artery; N_0_: no regional lymph node metastasis; N_1_: regional lymph node metastasis; M_0_: no distant metastasis; M_1_: distant metastasis). The current study was conducted in accordance with the Declaration of Helsinki. Note that the requirement for informed consent was waived by the board, as the study utilized data extracted from the SEER database.

**Fig 1:**
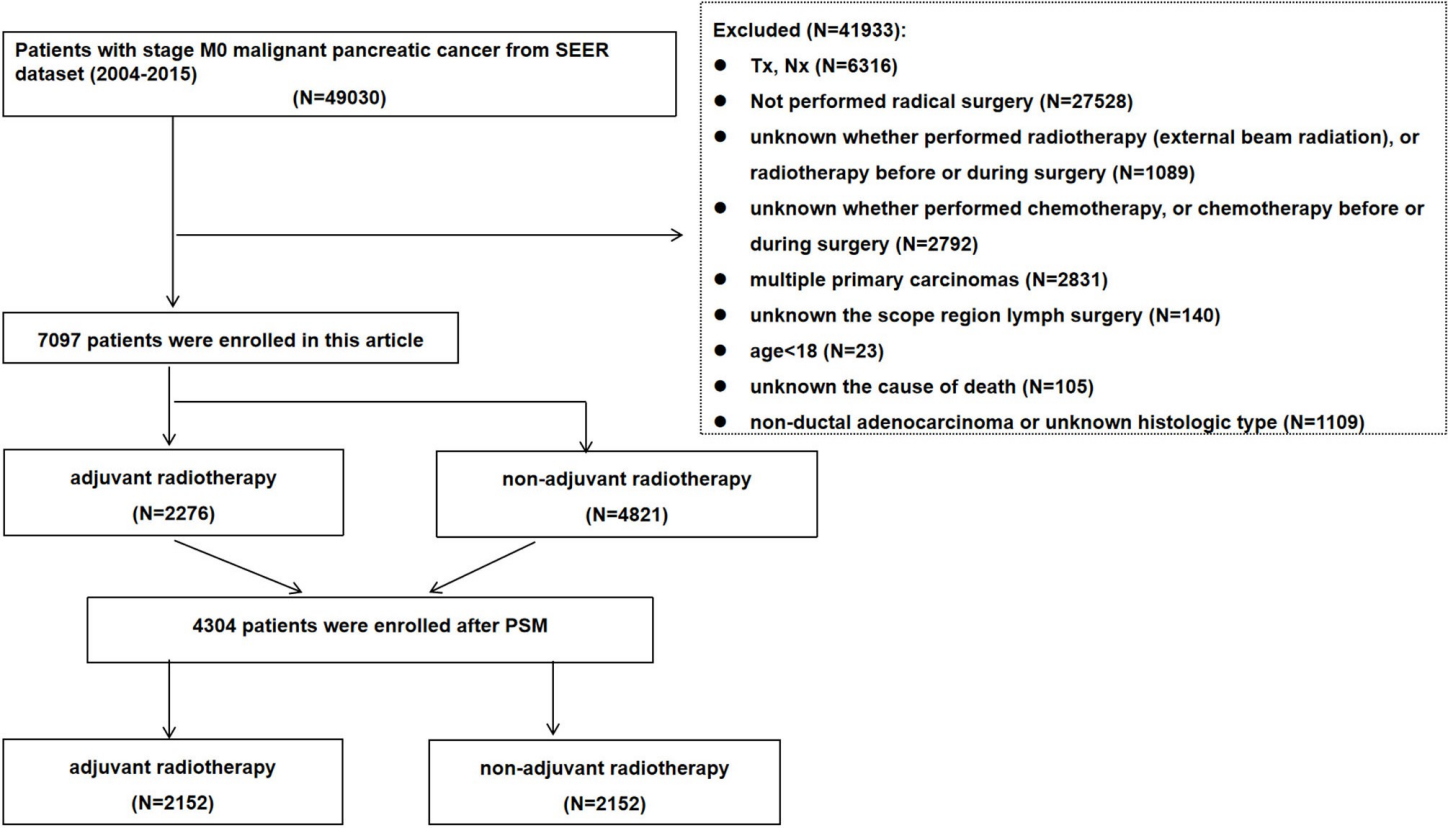
Flow chart of study enrollment and exclusions.

The inclusion criteria were as follows: patients diagnosed with M0 stage pancreatic cancer between 2004 and 2015. The exclusion criteria were as follows: (1) unknown T and N stage information; (2) patients without radical surgical treatment; (3) unknown whether performed radiotherapy or radiotherapy before or during surgery; received radiotherapy other than beam radiation; (4) unknown whether performed chemotherapy, or chemotherapy before or during surgery; (5) multiple primary carcinomas; (6) unknown the scope region lymph surgery; (7) age < 18; (8) unknown the cause of death; (9) non-ductal adenocarcinoma or unknown histological type (**Fig 1**).

### 2.2 Definition of endpoints

The endpoints of the current study were overall survival (OS) and cancer-specific survival (CSS). OS was defined as the interval from the time patients were diagnosed with pancreatic cancer to the time of death from any cause. CSS was defined as the interval from the time patients were diagnosed with pancreatic to the time of death caused by cancer.

### 2.3 Statistical analysis

All statistical analyses were conducted using SPSS 25.0. All the variables were converted to categorical variables. The differences in these variables between the radiotherapy group and the non-radiotherapy group were evaluated using a chi-square test. Univariate and multivariate COX proportional hazard models were used to identify prognostic factors that influenced survival outcome. The survival outcomes of the two groups were compared using the log-rank test, and the survival curves were plotted using the Kaplan-Meier method.

To reduce selection bias and balance the baseline characteristics between the two groups, we conducted propensity score matching (PSM). All variables were included in the PSM analysis. A 1:1 matching ratio was used, with an optimal caliper of 0.02. Before PSM, 7097 pancreatic cancer patients were enrolled in this study. After PSM, a total of 4304 patients were included in the analysis. Subgroup survival analysis was conducted in the matched population. P values less than 0.05 were considered statistically significant.

## 3.Results

### 3. 1 Characteristics of patients

Before PSM, 7097 resectable pancreatic cancer patients were enrolled in this study. Among them, 2276 patients received adjuvant radiotherapy (beam radiation), 4821 did not receive adjuvant radiotherapy, 4673 received adjuvant chemotherapy, 2424 did not receive adjuvant chemotherapy, 2154 received adjuvant chemoradiotherapy, and 2302 received none of them. Most patients were white (80.91%) and aged over 60 years (70.34%). Additionally, 61.46% of patients were married, 53.05% had a median income over 65,000 USD, and 50.13% were men. Years of diagnosis between 2011 and 2015 accounted for 54.35%, and most of the tumors occurred in the head of the pancreas (72.89%), 76.61% were T_3_ stage, and 64.75% were N_1_ stage. All the patients received radical pancreatic cancer surgery. Age at diagnosis, marital status, sex, year of diagnosis, primary site of tumor location, T stage, N stage, and chemotherapy were unbalanced between the two groups (all p < 0.05).

After PSM, a total of 4,304 resectable pancreatic cancer patients were included in this study. 2,152 of these patients received adjuvant radiotherapy, while the other 2,152 did not. Additionally, 4,060 patients were administered adjuvant chemotherapy, compared to 244 who did not receive. A subset of 2,030 patients underwent both adjuvant chemotherapy and radiotherapy, while 122 patients did not receive either. The characteristics that shows a significant imbalance and remained unbalanced post-PSM, including N stage, and scope region lymph surgery, were statistically significant (all p < 0.05) as shown in the following **Table 1**.

**Table 1:** Clinicopathological characteristics of patients.

Characteristic	Before PSM				After PSM			
	N = 7097	Non- Radiotherapy (N = 4821)	Radiotherapy (N = 2276)	P value	N = 4304	Non- Radiotherapy (N = 2152)	Radiotherapy (N = 2152)	P value
Race				0.644				0.615
White	5742	3890	1852		3467	1724	1743	
Black	711	483	228		462	241	221	
Other	644	448	196		375	187	188	
Age				< 0.001				0.427
≤60	2105	1270	835		1465	735	730	
60–70	2419	1571	848		1621	792	829	
>70	2573	1980	593		1218	625	593	
Marital status				< 0.001				0.899
Married	4362	2886	1476		2782	1389	1393	
Others	2735	1935	800		1522	763	759	
Median Income				0.079				0.329
≤ 65,000 USD	3332	2229	1103		2094	1063	1031	
> 65,000 USD	3765	2592	1173		2210	1089	1121	
Sex				0.001				0.855
Male	3558	2354	1204		2248	1121	1127	
Female	3539	2467	1072		2056	1031	1025	
Year of diagnosis				< 0.001				0.112
2004–2010	3240	2092	1148		1996	972	1024	
2011–2015	3857	2729	1128		2308	1180	1128	
Primary site				0.005				0.227
Head	5173	3459	1714		3199	1582	1617	
Body and tail	1226	875	351		718	380	338	
Others	698	487	211		387	190	197	
Tumor stage^a^				< 0.001				0.071
T_1_	493	399	94		225	131	94	
T_2_	900	639	261		509	257	252	
T_3_	5437	3612	1825		3404	1678	1726	
T_4_	267	171	96		166	86	80	
Nodal status^a^				< 0.001				< 0.001
N_0_	2502	1828	674		1384	756	628	
N_1_	4595	2993	1602		2920	1396	1524	
Scope region lymph Surgery^b^				0.170				< 0.001
0–3	576	406	170		444	288	156	
≥ 4	6521	4415	2106		3860	1864	1996	
Chemotherapy				< 0.001				1.000
Yes	4673	2519	2154		4060	2030	2030	
No	2424	2302	122		244	122	122	

Abbreviations: PSM, propensity score matching.

^a^Tumor stage and nodal status according to the Sixth Edition of the American Joint Committee on cancer (AJCC) staging manual.

^b^the procedure of removal, biopsy, or aspiration of regional lymph nodes performed during the initial work-up or first course of therapy at all facilities.

### 3.2 Predictors of OS and CSS before PSM

The variables were significantly correlated with survival outcome in the univariable Cox regression, including race, age, marital status, median income, year of diagnosis, primary site of tumor location, T stage, N stage, chemotherapy and radiotherapy (all p < 0.05). All variables from the univariable Cox regression were included in the multivariable Cox regression. In the multivariable Cox regression analysis, race, age, median income, sex, year of diagnosis, T stage, N stage, scope region lymph surgery, chemotherapy, and radiotherapy were independent predictors for OS of all patients. White, young age of onset, median income over 65,000 USD, female, year of diagnosis between 2011 and 2015, T_1_ stage, N_0_ stage, scope region lymph surgery ≥ 4, and received adjuvant chemotherapy and radiotherapy indicated better survival. Furthermore, adjuvant radiotherapy was associated with better survival compared to no-radiotherapy group in univariable Cox regression [no-radiotherapy versus radiotherapy (hazard ratio [HR], 1.24; 95% CI, 1.18 to 1.31; p < 0.01)], and in multivariable Cox regression, adjuvant radiotherapy also showed obvious survival benefit compared with no-radiotherapy [no-radiotherapy versus radiotherapy (HR, 1.20; 95% CI, 1.13 to 1.27; p < 0.01)] (**Table 2**).

**Table 2:** Univariate and multivariate analysis of overall survival.

Characteristic	Univariate analysis		Multivariate analysis	
	HR(95% CI)	P value	HR(95% CI)	P value
Race		< 0.001		0.003
White				
Black	1.068(0.982–1.162)	0.122	1.105(1.015–1.204)	0.022
Other	0.847(0.774–0.928)	< 0.001	0.899(0.820–0.986)	0.023
Age		< 0.001		< 0.001
≤ 60				
60–70	1.061(0.995–1.132)	0.070	1.065(0.998–1.136)	0.056
> 70	1.356(1.274–1.443)	< 0.001	1.283(1.204–1.368)	< 0.001
Marital status		< 0.001		0.104
Married				
Others	1.096(1.041–1.154)		1.046(0.991–1.103)	
Median income		< 0.001		< 0.001
≤ 65,000 USD				
> 65,000 USD	0.883(0.839–0.928)		0.891(0.847–0.938)	
Sex		0.157		0.013
Male				
Female	0.964(0.917–1.014)		0.936(0.888–0.986)	
Year of diagnosis		< 0.001		< 0.001
2004–2010				
2011–2015	0.877(0.833–0.922)		0.858(0.814–0.903)	
Primary site		< 0.001		0.252
Head				
Body and tail	0.846(0.790–0.907)	< 0.001	0.979(0.912–1.050)	0.550
Others	0.983(0.903–1.071)	0.697	1.065(0.977–1.161)	0.152
Tumor stage^a^		< 0.001		< 0.001
T_1_				
T_2_	1.647(1.438–1.887)	< 0.001	1.574(1.373–1.805)	< 0.001
T_3_	2.467(2.193–2.775)	< 0.001	2.239(1.980–2.531)	< 0.001
T_4_	4.361(3.688–5.155)	< 0.001	3.862(3.254–4.584)	< 0.001
Nodal status^a^		< 0.001		< 0.001
N_0_				
N_1_	1.726(1.634–1.823)		1.694(1.596–1.798)	
Scope region lymph Surgery^b^		0.716		< 0.001
0–3				
≥ 4	0.983(0.896–1.078)		0.838(0.761–0.921)	
Radiotherapy		< 0.001		< 0.001
Yes				
No	1.240(1.175–1.309)		1.197(1.128–1.270)	
Chemotherapy		< 0.001		< 0.001
Yes				
No	1.451(1.377–1.530)		1.541(1.452–1.635)	

Abbreviations: HR, hazard ratio.

^a^Tumor stage and nodal status according to the Sixth Edition of the American Joint Committee on cancer (AJCC) staging manual.

^b^the procedure of removal, biopsy, or aspiration of regional lymph nodes performed during the initial work-up or first course of therapy at all facilities.

According to the results of univariable Cox analysis for CSS, significant differences were observed in all variables except for sex and scope region lymph surgery. Considering sex and and scope region lymph surgery are independent predictors for OS, the two variables were incorporated along with other factors. Multivariable Cox regression analysis for CSS showed that the factors such as young age of onset, median income over 65,000 USD, year of diagnosis between 2011 and 2015, T_1_ stage, N_0_ stage, scope region lymph surgery ≥ 4, and received adjuvant chemotherapy and radiotherapy were associated with improved survival. Moreover, adjuvant radiotherapy demonstrated superior survival outcomes compared to the no-radiotherapy group in both univariable Cox regression analysis [no-radiotherapy versus radiotherapy (HR, 1.21; 95% CI to 1.15 to 1.28; p < 0.01)], and multivariable Cox regression analysis [no-radiotherapy versus radiotherapy (HR, 1.20; 95% CI, 1.13 to 1.28; p < 0.01)] (**Table 3**).

**Table 3:** Univariate and multivariate analysis of cancer-specific survival.

Characteristic	Univariate analysis		Multivariate analysis	
	HR(95% CI)	P value	HR(95% CI)	P value
Race		0.003		0.039
White				
Black	1.050(0.962–1.147)	0.276	1.078(0.985–1.180)	0.101
Other	0.860(0.782–0.945)	0.002	0.916(0.832–1.007)	0.070
Age		< 0.001		< 0.001
≤ 60				
60–70	1.044(0.977–1.116)	0.202	1.047(0.980–1.120)	0.175
> 70	1.285(1.204–1.371)	< 0.001	1.220(1.141–1.304)	< 0.001
Marital status		0.005		0.242
Married				
Others	1.080(1.023–1.140)		1.034(0.978–1.094)	
Median income		< 0.001		< 0.001
≤ 65,000 USD				
> 65,000 USD	0.886(0.841–0.934)		0.891(0.845–0.940)	
Sex		0.284		0.074
Male				
Female	0.972(0.922–1.024)		0.952(0.901–1.005)	
Year of diagnosis		< 0.001		< 0.001
2004–2010				
2011–2015	0.875(0.830–0.923)		0.855(0.810–0.902)	
Primary site		< 0.001		0.260
Head				
Body and tail	0.831(0.773–0.893)	< 0.001	0.970(0.901–1.044)	0.413
Others	0.972(0.889–1.063)	0.535	1.060(0.969–1.160)	0.204
Tumor stage^a^		< 0.001		< 0.001
T_1_				
T_2_	1.751(1.512–2.028)	< 0.001	1.649(1.422–1.913)	< 0.001
T_3_	2.693(2.369–3.062)	< 0.001	2.383(2.086–2.722)	< 0.001
T_4_	4.849(4.061–5.788)	< 0.001	4.201(3.505–5.036)	< 0.001
Nodal status^a^		< 0.001		< 0.001
N0				
N1	1.816(1.714–1.925)		1.761(1.654–1.875)	
Scope region lymph Surgery^b^		0.584		< 0.001
0–3				
≥ 4	0.973(0.884–1.072)		0.812(0.735–0.897)	
Radiotherapy		< 0.001		< 0.001
Yes				
No	1.211(1.145–1.281)		1.199(1.127–1.275)	
Chemotherapy		< 0.001		< 0.001
Yes				
No	1.388(1.313–1.467)		1.498(1.407–1.594)	

Abbreviations: HR, hazard ratio.

^a^Tumor stage and nodal status according to the Sixth Edition of the American Joint Committee on cancer (AJCC) staging manual.

^b^the procedure of removal, biopsy, or aspiration of regional lymph nodes performed during the initial work-up or first course of therapy at all facilities.

### 3.3 Survival outcomes and subgroup analysis after PSM

**Fig 2:**
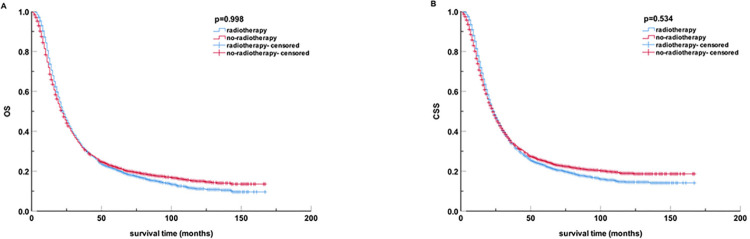
Kaplan-Meier curves for OS and CSS of all patients after PSM. (A) OS; (B) CSS. PSM, propensity score matching; OS, overall survival; CSS, cancer-specific survival.

After PSM, the mOS and mCSS of pancreatic cancer patients were 23 months (95% CI, 21.86 to 24.14) and 24 months (95% CI, 22.73 to 25.27), respectively, in the radiotherapy group. While in the non-radiotherapy group, they were 22 months (95% CI, 20.77 to 23.23) and 23 months (95% CI, 21.58 to 24.42), respectively. No statistically significant difference was observed between the two groups regarding OS and CSS characteristics (all p >0 .05) as shown in **Fig 2**.

**Fig 3:**
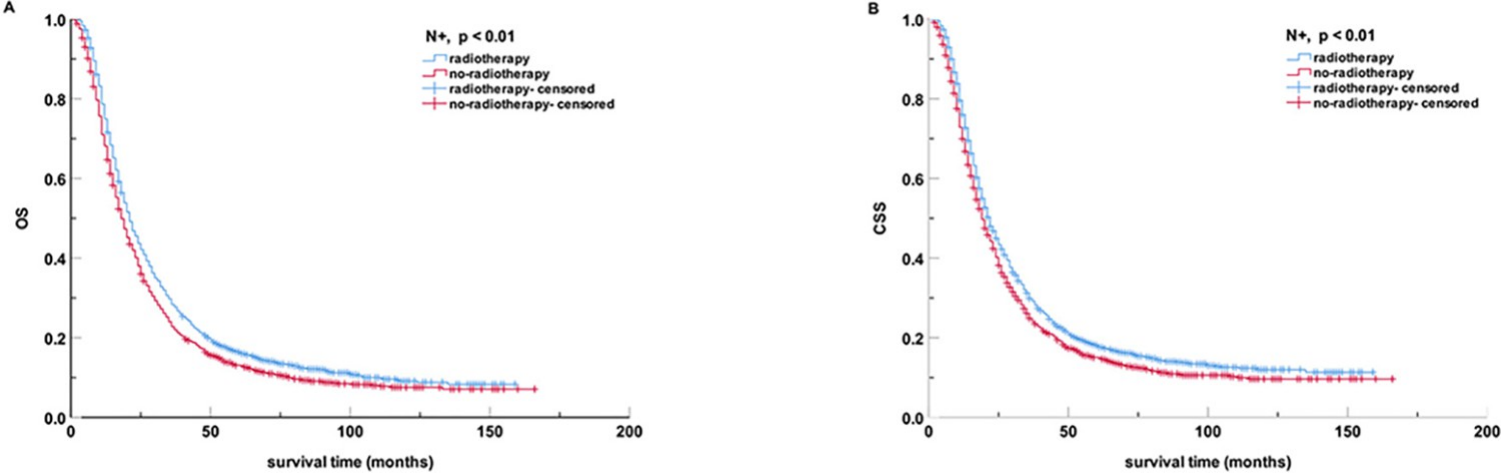
Kaplan-Meier curves for OS and CSS of N+ subgroup patients after PSM. (A) OS; (B) CSS. PSM, propensity score matching; OS, overall survival; CSS, cancer-specific survival.

In the subgroup analysis, regardless of age, marriage, median income, sex, year of diagnosis, or received chemotherapy, radiotherapy did not improve survival of OS (all p > 0.05). The patients in the radiotherapy group with the N_0_ stage (N-) exhibited a higher all-cause mortality rate and cancer-specific mortality rate compared to the patients without radiotherapy (all p < 0.01) (**Table 4**). However, among patients with N_1_ stage (N+), radiotherapy reduced the all-cause mortality rate and cancer-specific mortality rate when compared to no radiotherapy (all p < 0.01, **Fig 3**). The mOS for radiotherapy and non-radiotherapy were 21 months (95% CI, 19.82 to 22.18) and 18 months (95% CI, 16.88 to 19.12), respectively.

**Table 4:** Survival analysis by Kaplan-Meier method in patients after PSM.

Groups	Receive radiotherapy	N	mOS(95% CI, months)	P value	mCSS(95% CI, months)	P value
All	Yes	2152	23(21.857–24.143)	0.998	24(22.727–25.273)	0.534
	No	2152	22(20.768–23.232)		23(21.584–24.416)	
N_0_	Yes	628	28(24.613–31.387)	0.001	30(26.400–33.600)	< 0.001
	No	756	34(29.472–38.528)		41(33.682–48.318)	
N_1_	Yes	1524	21(19.816–22.184)	< 0.001	22(20.790–23.210)	< 0.001
	No	1396	18(16.878–19.122)		19(17.807–20.193)	
Head	Yes	1617	23(21.750–24.250)	0.278	24(22.634–25.366)	0.446
	No	1582	21(19.736–22.264)		22(20.674–23.326)	
Body and tail	Yes	338	23(19.321–26.679)	0.019	25(21.153–28.847)	0.010
	No	380	29(24.369–33.631)		32(27.593–36.407)	
T_1_	Yes	94	36(21.836–50.164)	0.005	36(13.066–58.934)	0.001
	No	131	113(64.081–161.919)		104(92.562–115.877)	
T_2_	Yes	252	31(26.718–35.282)	0.392	32(28.225–35.775)	0.233
	No	257	31(26.198–35.802)		33(24.871–41.129)	
T_3_	Yes	1726	22(20.791–23.209)	0.147	23(21.693–24.307)	0.357
	No	1678	20(18.768–21.232)		21(19.700–22.300)	
T_4_	Yes	80	14(9.617–18.383)	0.012	16(11.938–20.062)	0.015
	No	86	11(8.985–13.015)		11(8.905–13.095)	
Male	Yes	1127	22(20.555–23.445)	0.912	23(21.305–24.695)	0.679
	No	1121	22(20.375–23.625)		23(21.382–24.618)	
Female	Yes	1025	23(21.117–24.883)	0.890	24(22.041–25.959)	0.645
	No	1031	22(20.116–23.884)		24(21.536–26.464)	
Age ≤ 60	Yes	730	24(21.900–26.100)	0.435	24(21.883–26.117)	0.461
	No	735	22(19.911–24.089)		23(20.936–25.064)	
Age 60–70	Yes	829	23(21.268–24.732)	0.485	24(21.979–26.021)	0.464
	No	792	25(23.053–26.947)		26(24.010–27.990)	
Age > 70	Yes	593	21(19.044–22.956)	0.751	22(19.639–24.361)	0.200
	No	625	18(16.149–19.851)		21(18.574–23.426)	
Chemotherapy	Yes	2030	23(21.767–24.233)	0.542	24(22.641–25.359)	0.972
	No	2030	22(20.768–23.232)		23(21.594–24.406)	
Non-chemotherapy	Yes	122	17(13.994–20.006)	0.081	19(16.000–22.000)	0.041
	No	122	16(7.956–24.044)		19(11.265–26.735)	
Married	Yes	1393	22(20.599–23.401)	0.428	23(21.404–24.596)	0.301
	No	1389	23(21.410–24.590)		24(22.200–25.800)	
No-married	Yes	759	24(22.023–25.977)	0.274	24(21.891–26.109)	0.712
	No	763	20(18.058–21.942)		22(19.893–24.107)	
Income ≤ 65,000 USD	Yes	1031	21(19.768–22.232)	0.113	21(19.664–22.336)	0.147
	No	1063	19(17.449–20.551)		20(18.387–21.613)	
Income > 65,000 USD	Yes	1121	25(23.134–26.866)	0.108	26(24.002–27.998)	0.017
	No	1089	24(21.890–26.110)		26(23.610–28.390)	
Year of diagnosis: 2004–2010	Yes	1024	21(19.622–22.378)	0.984	22(20.456–23.544)	0.885
	No	972	19(17.371–20.629)		20(18.230–21.770)	
Year of diagnosis: 2011–2015	Yes	1128	24(22.150–25.850)	0.851	25(23.110–26.890)	0.605
	No	1180	24(22.198–25.802)		26(24.076–27.924)	

Abbreviations: PSM, propensity score matching; mOS, median overall survival; mCSS, median cancer-specific survival.

For patients in the radiotherapy group with pancreatic head cancer, the all-cause mortality rate and cancer-specific mortality rate showed no significant difference compared to those not receiving radiotherapy (p = 0.28 and p = 0.45, respectively). Among the patients with pancreatic body and tail cancer, those in the radiotherapy group exhibited a higher all-cause mortality rate and cancer-specific mortality rate than the patients in the non-radiotherapy group (p = 0.02 and p = 0.01, respectively). Patients with T_1_ stage in the radiotherapy group exhibited higher all-cause mortality and cancer-specific mortality rates compared to the non-radiotherapy group (p = 0.01 and p < 0.01, respectively). In contrast, patients with the T_4_ stage who received radiotherapy had lower all-cause mortality rates and cancer-specific mortality rate than the non-radiotherapy group (p = 0.01 and p = 0.02, respectively). Radiotherapy tended to shorten survival of those patients in T_2_ stage and prolong the survival in T_3_ stage. However, there were no significant difference (all p > 0.05). Compared with chemotherapy, radiochemotherapy did not bring benefit to OS and CSS of pancreatic cancer patients (all p > 0.05). In no-chemotherapy group, median OS was 17 months (95%CI, 13.99 to 20.01), 16 months (95%CI, 7.96 to 24.04) for patients undergone radiotherapy, non-radiotherapy, respectively (p = 0.08). And median CSS was 19 months (95%CI, 16.000 to 22.000), 19 months (95%CI, 11.27 to 26.74) for patients undergone radiotherapy, non-radiotherapy, respectively (p = 0.04, **Table 4**).

### 3.4 Subgroup analysis of survival in lymph node positive patients

Further exploration was conducted on the prognostic impact of radiotherapy on various subgroups of stage N_1_ (N+) patients. The result showed a survival benefit of radiotherapy among patients with tumors in the head of the pancreas (all p < 0.01), exhibiting a mOS of 22 months versus 19 months and a mCSS of 22 months versus 19 months. In contrast, no significantly difference was noted in the body and tail of pancreatic cancer (p = 0.82 and p = 0.52, respectively). For the T stage subgroup, radiotherapy brought benefit in the T_2_ stage group (p = 0.03 and p = 0.06, respectively), T_3_ stage group (all p < 0.01) and T_4_ stage group (all p = 0.01), while radiotherapy brought no benefit in T_1_ stage group (p = 0.88 and p = 0.47, respectively). Moreover, the results revealed that both male and female patients with node-positive pancreatic cancer could benefit from radiotherapy, regardless of their gender (p < 0.05). OS and CSS for patients receiving adjuvant radiotherapy was longer than no-radiotherapy in the age ≤ 60 group (all p < 0.01). As for the age 60–70 group and age > 70 group, there was no OS benefit of radiotherapy compared with no-radiotherapy (all p = 0.058). Note that, for the chemotherapy group, radiotherapy also reduced the all-cause and cancer-specific mortality rates (all p < 0.01). But for the no-chemotherapy group, there was no OS and CSS benefit of radiotherapy compared with no-radiotherapy (p = 0.85 and p = 0.39, respectively). The median overall survival time and cancer-specific survival time of N+ group patients were shown in **Table 5**.

**Table 5:** Median OS and CSS time of N+ group patients.

Groups	Receive radiotherapy	N	mOS(95% CI, months)	P value	mCSS(95% CI, months)	P value
Head	Yes	1201	22(20.702–23.298)	< 0.001	22(20.605–23.395)	< 0.001
	No	1099	19(17.760–20.240)		19(17.690–20.310)	
Body and tail	Yes	194	19(16.978–21.022)	0.815	20(16.878–23.122)	0.522
	No	187	20(16.795–23.205)		22(17.344–26.656)	
T_1_	Yes	35	21(18.102–23.898)	0.880	21(18.102–23.898)	0.471
	No	34	27(20.155–33.845)		27(17.014–36.986)	
T_2_	Yes	142	29(23.832–34.168)	0.030	30(24.390–35.610)	0.061
	No	107	21(17.876–24.124)		22(18.927–25.073)	
T_3_	Yes	1291	21(19.678–22.322)	0.001	21(19.654–22.346)	0.004
	No	1202	18(16.786–19.214)		19(17.704–20.296)	
T_4_	Yes	56	18(15.252–20.748)	0.007	18(15.252–20.748)	0.012
	No	53	10(8.415–11.585)		11(8.664–13.336)	
Male	Yes	813	21(19.439–22.561)	0.007	21(19.431–22.569)	0.026
	No	727	18(16.487–19.513)		20(18.316–21.684)	
Female	Yes	711	22(20.184–23.816)	0.002	22(19.847–24.153)	0.005
	No	669	18(16.444–19.556)		19(17.308–20.692)	
Age ≤ 60	Yes	527	21(19.017–22.983)	0.002	22(19.893–24.107)	0.003
	No	487	18(16.663–19.337)		18(16.445–19.555)	
Age 60–70	Yes	597	21(19.100–22.900)	0.058	22(19.935–24.065)	0.042
	No	504	21(18.933–23.067)		22(20.025–23.975)	
Age > 70	Yes	400	20(17.943–22.057)	0.058	20(17.654–22.346)	0.392
	No	405	16(13.916–18.084)		18(15.786–20.214)	
Chemotherapy	Yes	1449	21(19.795–22.205)	< 0.001	22(20.677–23.323)	< 0.001
	No	1379	18(16.884–19.116)		19(17.806–20.194)	
Non-chemotherapy	Yes	75	15(10.285–19.715)	0.854	17(12.004–21.996)	0.385
	No	17	12(6.903–17.097)		22(0.000–44.994)	

Abbreviations: mOS, median overall survival; mCSS, median cancer-specific survival; N+, lymph node positive.

## 4. Discussion

The incidence of pancreatic cancer is increasing every year, and its prognosis is so poor [12]. Tumor recurrence is common in pancreatic cancer after surgery [13,14], thus, the need for adjuvant comprehensive radiation therapy is essential to improve the patient survival rate. Previous randomized controlled trials and retrospective studies have reported inconsistent results regarding the effect of adjuvant radiotherapy on pancreatic cancer after radical surgery [15–17]. They are susceptible to bias due to numbers of insufficient cases and the lack of detailed screening for radiotherapy benefits in certain retrospective studies. Hence, this study was initiated to examine the role of radiotherapy in pancreatic cancer after up-front radical surgery within a substantial cohort of 7097 observations, aiming to identify which patient could benefit from radiotherapy and which do not.

Significant differences were observed in the patient populations who received adjuvant radiation [18]. Specifically, patients in the radiotherapy group were more likely to be young, married, male, 2004–2010 diagnosed, T_3-4_ stage, N_1_ stage, head of the pancreas, and received chemotherapy. Our study demonstrated that younger(age < 60 years), median income greater than 65,000 USD, female, year of diagnosis between 2011 and 2015, T_1_ stage, N_0_ stage, scope region lymph surgery ≥ 4, and received adjuvant chemotherapy and radiotherapy were associated with better survival, which was in accordance with previously published findings [19]. Our research indicated that adjuvant radiotherapy brought survival benefits to pancreatic cancer patients after up-front radical surgery before PSM.

After PSM, the KM analysis showed little significant difference that radiotherapy benefits the survival of pancreatic cancer patients after upfront radical surgery. A detailed analysis of the survival curve showed that radiotherapy could offer a survival benefit in the early-stage, while adjuvant radiotherapy increased the risk of mortality in the later stages. This outcome could be related to various factors including late toxicity of radiotherapy, distant metastasis, and other confounders [20]. The analysis led to the hypothesis that while some patients may benefit from adjuvant radiotherapy, others may not, which was confirmed through further subgroup analyses. Subgroup analysis suggested that radiotherapy significantly reduced the risk of death in patients within stage N_1_ (N+) and T_4_ groups, while adjuvant radiotherapy posed a risk of death for patients in stage T_1_, N_0_, those with tumors in the body and tail of pancreas. When comparing the above findings with other studies, it is important to note that the positive results align while the negative results vary. A retrospective study identified a significant survival advantage for the use of adjuvant radiotherapy over surgery alone or neoadjuvant radiotherapy in treating stage II (T_3_N_0_, T_1-3_N_1_) pancreatic cancer. Radiotherapy was not associated with survival benefit in stage I (T_1-2_N_0_) patients [21]. Meanwhile, another analysis of 10,097 patients with pancreatic adenocarcinoma cancer showed that the survival benefit of adjuvant chemoradiotherapy was more significant in patients with female or T3 or lymph nodes positive [22]. Due to different screening schemes, the results could assist in better identifying individual suitability for choosing adjuvant radiotherapy and who should be cautious about selecting radiotherapy in clinical practice.

Previous research has indicated that adjuvant radiotherapy enhanced survival in resectable pancreatic cancer patients with N_1_ stage (N+), aligning with established findings [23–25]. However, the subgroup analysis of the adjuvant radiotherapy in N_1_ stage (N+) pancreatic cancer patients has not been demonstrated. Then, the further analysis of our study found that not all patients with N+ stage could benefit from adjuvant radiotherapy. Notably, patients with tumors located in the head of the pancreas could experience survival benefits, while those with tumors in the body and tail did not. The high metabolic activity of the body and tail of the pancreas, along with the extensive target area for postoperative radiotherapy and its associated significant side effects, counterbalance the potential benefits offered by adjuvant radiotherapy. This observation reflects the different biological behaviors of pancreatic cancer across anatomical sites [19]. A study of pancreatic cancer patients identified a robust gene expression signature associated with tumor location, suggesting that head, body and tail of pancreatic cancer were different [26]. Pancreatic body and tail cancers often present with distant metastases more than pancreatic head cancers [27]. Postoperative local control is less important for pancreatic body and tail cancers than for pancreatic head cancers. Thus, pancreatic cancer is usually treated according to the location. However, tumor location was not an independent prognostic factor for pancreatic cancer after adjusting for potential confounders, which is in line with previous findings [28,29].

The significance of T stage in relation to adjuvant radiotherapy is underscored by the discovery that patients with T_2-4_N+ stage could potentially benefit from postoperative radiotherapy, whereas those with T_1_N+ stage did not demonstrate such benefits. The addition of adjuvant radiotherapy did not confer any benefit to T_1_N_1_ patients, despite its association with a high R0 removal rate in early-stage patients. However, it was found to increase the incidence of radiotherapy toxicity [30]. This result diverge from some studies that suggest postoperative adjuvant radiotherapy has a better prognosis, and adjuvant radiotherapy is also preferred for pancreatic cancer patients with stage T_1_N_1_M_0_ [31]. This result potentially due to the lack of comprehensive subgroup analyses across different T stages in prior research. Instead, additional studies on neoadjuvant chemoradiotherapy have been added. Radiotherapy was found to benefit N+ stage patients irrespective of sex. Meanwhile, Age is the reference factor to guide the selection of radiotherapy for patients with stage IIB/III pancreatic cancer [32]. As for the age factor, our study showed that all N+ stage patients could benefit from adjuvant radiotherapy except for the the age > 60 groups. This result suggests that radiotherapy for age > 60 patients should be approached with caution due to increased risks. We speculate that this might be due to lack of standard treatment regimens and dosages of radiotherapy. The elderly might not receive standard chemotherapy at the time of radiotherapy [33]. Compared to chemotherapy alone, chemoradiotherapy significantly improved the outcome for N+ stage patients. For the patients in the non-chem group, however, radiotherapy did not bring better survival. This finding could be explained that adjuvant chemotherapy plays an crucial role in postoperative pancreatic cancer. Chemotherapy is the cornerstone of postoperative adjuvant therapy for resectable pancreatic cancer [34].

The study presents several limitations. It is retrospective in nature, which could introduce selectivity bias in the data collection process. Patients were diagnosed as pancreatic cancer according to the Sixth Edition of the AJCC staging manual, which is slightly different from the eighth edition staging currently used. Moreover, all the data were exclusively collected from the SEER database, which lacked detailed chemotherapy and radiotherapy protocol, incisal edge information, and physical fitness scores. To enhance the robustness of these concerns, it is recommended that further clinical trials should be undertaken on the following issues: (1) With the advancement of precision radiotherapy, there is a growing need to further explore the radiotherapy target area and dose fraction for postoperative radiotherapy in pancreatic cancer. This exploration should be based on improved effectiveness and safety, aiming to enhance its role in postoperative adjuvant therapy for pancreatic cancer. (2) To investigate the feasibility of incorporating postoperative adjuvant radiotherapy in specific subgroups where achieving local control can lead to survival benefits.

## 5. Conclusion

The analysis demonstrates that N_1_ stage (N+) pancreatic cancer patients who have received the up-front radical surgery with T_2-4_ stage, primary focus on the head of the pancreas, young age of onset, and with combination of chemotherapy may benefit from adjuvant radiotherapy. In contrast, radiotherapy should be approached with caution for patients at the T_1_ stage and N_0_ stage (N-), especially when the tumor is located on the body and tail of the pancreas. These insights may contribute to the development of individualized adjuvant radiotherapy for pancreatic cancer patients following up-front radical surgery.
